# Associations between Community Environmental-Level Factors and Diet Quality in Geographically Isolated Australian Communities

**DOI:** 10.3390/ijerph16111943

**Published:** 2019-05-31

**Authors:** Thomas P. Wycherley, Jolieke C. van der Pols, Mark Daniel, Natasha J. Howard, Kerin O’Dea, Julie K. Brimblecombe

**Affiliations:** 1 Alliance for Research in Exercise, Nutrition and Activity, University of South Australia, Adelaide 5000, Australia; 2 Wellbeing and Preventable Chronic Diseases Division, Menzies School of Health Research; Darwin 0810, Australia; julie.brimblecombe@monash.edu; 3 School of Exercise and Nutrition Sciences, Queensland University of Technology, Brisbane 4001, Australia; j.vanderpols@qut.edu.au; 4 Health Research Institute, University of Canberra, Canberra, 2617, Australia; mark.daniel@canberra.edu.au; 5 Department of Medicine, St Vincent’s Hospital, The University of Melbourne, Fitzroy 3010, Australia; 6 Wardliparingga Aboriginal Health Equity Theme, South Australian Health and Medical Research Institute, Adelaide 5000, Australia; Natasha.Howard@unisa.edu.au; 7 School of Health Sciences, University of South Australia, Adelaide 5000, Australia; kerin.odea@unisa.edu.au; 8 Department of Nutrition, Dietetics and Food, Monash University, Melbourne 3800, Australia

**Keywords:** cardio-metabolic health, Indigenous Australians, spatial epidemiology

## Abstract

Remote Indigenous Australians experience disproportionately poor cardio-metabolic health, which is largely underpinned by adverse dietary intake related to social determinants. Little evidence exists about the community environmental-level factors that shape diet quality in this geographically isolated population group. This study aimed to explore the modifiable environmental-level factors associated with the features of dietary intake that underpin cardio-metabolic disease risk in this population group. Community-level dietary intake data were estimated from weekly store sales data collected throughout 2012 and linked with concurrent social, built, and physical environmental dimension data for 13 remote Indigenous Australian communities in the Northern Territory. Statistical analyses were performed to investigate associations. At the community level, store sales of discretionary foods were lower in communities with greater distance to a neighbouring store (r = −0.45 (*p* < 0.05)). Sales of sugar-sweetened beverages were lower in communities with higher levels of household crowding (r = −0.55 (*p* < 0.05)), higher levels of Indigenous unemployment (r = −0.62 (*p* = 0.02)), and greater distance to neighbouring stores (r = −0.61 (*p* = 0.004)). Modifiable environmental-level factors may be associated with adverse diet quality in remote Indigenous Australian communities and further investigations of these factors should be considered when developing policies to improve dietary intake quality in geographically isolated populations.

## 1. Introduction

Indigenous Australians, who have adapted to extreme changes in their environment over millennia and previously experienced minimal chronic disease, now, because of colonization, experience a reduced life expectancy which can be largely attributed to a greater occurrence of cardio-metabolic disease [1]. Furthermore, a relatively higher occurrence of cardio-metabolic disease is experienced by those who live in geographically isolated locations [1]. Much of this cardio-metabolic disease burden can be attributed to poor nutrition across the life course of individuals, underpinned by social determinants such as food access and affordability [2]. This has resulted in a dietary profile that is typically higher in discretionary choices (i.e., foods or beverages high in saturated fat, added sugars, or salt [3]), in particular sugar-sweetened beverages (SSBs), and lower in fruits and vegetables (F&V) as compared to that of the general Australian population [4]. These dietary intake characteristics have been associated with poor cardio-metabolic health. Specifically, a high intake of discretionary foods has negative implications for weight status and cardio-metabolic health [5]. Several high-impact studies [6,7] have shown that the consumption of sugar-sweetened beverages is associated with excess calorie intake, weight gain, and a greater risk of type 2 diabetes and cardiovascular disease; in addition, a low intake of fruits and vegetables has been linked in cohort studies to a greater incidence of cardiovascular disease [8,9].

Relatively little empirical evidence exists on factors that shape the diet quality of geographically isolated populations, including Indigenous Australians living in remote communities [10]. The identification of modifiable environmental-level factors that are associated with dietary intake features that underpin cardio-metabolic disease risk in such communities would assist the development of policies to enhance nutrition and reduce the prevalence of chronic preventable disease, and provide much needed evidence to these communities to help inform advocacy and local-level policy. 

The aim of this study was to conduct a descriptive analysis to explore modifiable environmental-level factors that are associated with the features of dietary intake that underpin cardio-metabolic disease risk in geographically isolated Indigenous Australian communities. 

## 2. Materials and Methods

The current study analysed linked data from two previous studies; the Stores Healthy Options Project in Remote Indigenous Communities (SHOP@RIC) study [11] and the Environments and Remote Indigenous Cardio-Metabolic Health Project (EnRICH) [12]. Dietary intake estimate data for 20 remote Indigenous Australian communities in the Northern Territory were derived from the baseline period (49 weeks throughout 2012) of the SHOP@RIC study. The data were community-level (i.e., average population) dietary intake estimates obtained using store point-of-sale data. Community store point-of-sale data analysis is increasingly being used as a cost-effective means of estimating dietary intake for remote Indigenous Australian communities and provides powerful information that can be used by community groups to inform decision-making for improved health [13,14]. In brief, this method involves linking individual food and beverage products sold in the store, along with sales quantities, with their applicable Food Standards Australian and New Zealand product nutrition profile [13]. It is widely accepted that for remote Indigenous Australian communities, within-community food and beverage providers supply the majority of the residents’ dietary intake [13]. However, this method has a number of limitations, including the fact that food waste and externally purchased or traditional foods are not accounted for. We have recently reported that in three very remote Indigenous Australian communities the community store per se provided ~98% of the food and beverages that were derived from all food and beverage providers within the community (i.e., only ~2% was derived from other community-based food and beverage providers such as school breakfast programs) [15].

Comprehensive environmental-level factor data were obtained for 13 of the 20 SHOP@RIC study communities via linkage with the EnRICH Project. The EnRICH Project categorised community-level indicators, collected during a time period concurrent to the dietary intake data, representing the social, built, and physical environmental dimensions (e.g., Australian Bureau of Statistics 2011 Census data). The SHOP@RIC study collected information on several environmental-level factors, including frequency of food delivery and distance to the nearest community with a food retail store, which were used to provide a sample size of up to 20 for the analysis of these factors (frequency of food delivery information was not available for one community, four communities did not have specific distance to the nearest community data available due to being island communities or having no identifiable neighbouring community; these were coded as 500 km to derive the median for the dichotomized analyses).

Statistical analyses were conducted to investigate the associations between community modifiable environmental-level factors and the features of dietary intake that are relevant to cardio-metabolic disease risk. A Pearson correlation was used to examine the associations between continuous variables. Environmental-level factor variables were also dichotomised into equal-or-above or below median value categories and independent *t*-tests were conducted to investigate the differences between categories for dietary intake variables. Regression models for each dietary outcome were also performed in a supplementary analysis (Tables S1–S4). All observations were included for analyses using the environmental-level variables collected in the SHOP@RIC study (frequency of food delivery and distance to the nearest community with a food retail store). A separate analysis of these factors using just the 13 common communities from the SHOP@RIC and EnRICH studies was also performed (Table S5). Differences between the common communities in the SHOP@RIC and EnRICH studies and the non-common SHOP@RIC communities were evaluated in a supplementary analysis (Table S6).

The dietary intake variables of interest were the community-level percentage energy (%E) from discretionary foods [3], percentage energy from SSBs, and percentage dietary intake quantity from F&Vs. The percent of the estimated average requirement (%EAR) for energy was also investigated as a proxy for the amount of total food and drink consumed that was likely to be obtained through the community store. We assessed whether these dietary intake characteristics were associated with the distance from the community to the nearest (neighbouring) store (km), the frequency of food delivery to the store (days), the Australian Bureau of the Census measurement of percentage Indigenous unemployment, the median Indigenous household income ($/week), Indigenous household crowding (percentage dwellings requiring additional bedroom/s), and Indigenous education attainment (percentage Indigenous persons with educational attainment ≥school year nine), all expressed at the community level. 

Ethics approval for the SHOP@RIC study, including the present analyses, was granted by the Human Research Ethics Committees of the Northern Territory Department of Health and Menzies School of Health Research (HREC-2012-1711) and Central Australia (HREC-12-13). 

## 3. Results

Comprehensive linked data were obtained for 13 remote Indigenous Australian communities. The individual community populations ranged from 139–1079 persons (range of 125–1079 in all 20 SHOP@RIC communities). The 13 linked communities were similar to the non-linked SHOP@RIC communities for discretionary foods (%E) and fruits and vegetables (% quantity), but were lower for SSB (%E) and %EAR for energy (*p* < 0.01; Table S6). 

Detailed results are provided in Table 1. At the community level, the correlation coefficients indicate that sales of discretionary foods as a percent of the total energy were lower in communities with a greater distance to a neighbouring store (r = −0.45 (*p* < 0.05)). Sales of SSBs as a percent of the total energy were lower in communities with higher levels of household crowding (r = −0.55 (*p* < 0.05)), higher levels of Indigenous unemployment (r = −0.62 (*p* = 0.02)), and when the distance to a neighbouring store was greater (r = −0.61 (*p* < 0.01)). The percent of the estimated average requirement for energy obtained through the community store was higher in communities with a greater distance to a neighbouring store (r = 0.67 (*p* < 0.01)). No correlation was found between environmental-level factors and fruit and vegetable sales as a percent of total food and beverage sales quantity. No dietary intake variables were correlated with a community’s frequency of food delivery, Indigenous educational attainment, or the community average household income. 

For the dichotomised categorical analysis, communities with a distance to a neighbouring store ≥82 km had a 1.9% (absolute) lower SSB %E compared to those with a distance <82 km. Only 82.2% of the %EAR for energy was derived from the community store when the community distance to a neighbouring store was <82 km, compared to 106.4% for those having a distance ≥82 km. The percentage of total food purchased derived from F&V was 2.3% (absolute) lower for communities with an income ≥$1187/week compared to <$1187/week. No other dietary intake variables varied significantly according to the dichotomised categorical environmental-level factors.

The supplementary regression analyses (Supplementary Materials 1) did not indicate significant associations between any environmental-level factors and dietary factors (Tables S1–S3); however, the distance to a neighbouring store (km) and the frequency of food delivery were significantly associated with %EAR (*p* < 0.01). The supplementary analysis using only the 13 common communities from the SHOP@RIC and EnRICH studies for frequency of food delivery and distance to the nearest community with a food retail store found a statistically significant positive correlation between the distance to a neighbouring store and %EAR for energy (r = 0.80 (*p* = 0.01)). No other correlations or *t*-tests in the sub-analysis were significant (Table S5).

## 4. Discussion

This study identified several potentially modifiable environmental-level factors in remote Indigenous Australian communities that were associated with dietary intake features known to underpin cardio-metabolic disease. Factors identified as being associated with one or more markers of lower dietary intake quality included lesser household crowding, lower Indigenous unemployment, lower median Indigenous household income, and lesser distance to a neighbouring store. To our knowledge, this is the first study to attempt to explore the associations between community environmental-level factors and dietary intake estimates for remote Indigenous Australian communities. These findings affirm the tenet of health promotion practice to account for environmental factors when developing policies to improve dietary intake quality in disadvantaged population groups experiencing structural disadvantage [16].

The positive association between community distance to a neighbouring store and %EAR for energy derived from community store sales supports the notion that the more isolated a community is, the lower the amount of total resident-consumed food and beverage products purchased from outside the community. This emphasises the need for adequate stock and variety of nutritious foods in more isolated community stores. This association also suggests that sales data for communities closer to a neighbouring community are less likely to provide a complete dietary intake picture.

SSB sales as a percent of total energy were inversely associated with Indigenous unemployment and household crowding. Although the direction of these associations is opposite to that typically observed between SSB intake and socioeconomic factors [17], there are examples where a similar direction of association has been observed in countries undergoing socio-economic transition [18]. This observation suggests that SSBs may be treated as a luxury item in remote Indigenous Australian communities, being more likely to be consumed when money is relatively more available [19], although we must consider this interpretation with caution given the lack of association between SSB sales and household income. This concept may also provide some explanation for the higher amount of fruit and vegetables (% quantity) observed in communities with lower Indigenous household income (i.e., these items may be relatively de-prioritized in favour of discretionary products when money is relatively more available). Hence, in order to reduce SSB intake (and potentially also increase fruit and vegetable intake) in populations experiencing low affordability, such as remote Indigenous Australians, intervention approaches beyond those typically employed to increase fruit and vegetable intake and reduce the intake of cheap, energy dense, unhealthy foods associated with low food affordability may need to be considered [20]. Although it was beyond the scope of the current study, it is important to consider that the merchandising strategies and the internal environments of stores can also play an important role in influencing consumer choices [21].

There are several limitations to this study. Although the included communities represented different geographical areas and language groups, they were all single-store communities with a relatively small population size located only in the Northern Territory. Hence, to what extent these specific results are generalisable to other geographically isolated communities requires confirmation. The analysis was performed using aggregated community-level data and therefore does not consider individual-level variation within the communities. Due to the limited number of observations (statistical power), more complex regression analyses could not be effectively performed to investigate predictive relationships and the impact of confounders (Supplementary Materials 1). Furthermore, the low sample size was not conducive to enabling adjustments for multiple comparisons and our analysis is therefore prone to type 1 errors. Being able to include the additional non-common SHOP@RIC communities in the analyses for the frequency of food delivery and distance to the nearest community with a food retail store environmental-level factors enhanced the power of these analyses. However, that conventional statistical significance was no longer observed for several associations in the subset analysis of these factors using only the 13 common study communities (Supplementary Materials 2) highlights the limitations relating to sample size in the broader study. Despite methodological limitations relating to sample size, correlation coefficients were moderate-to-strong. Demonstrating associations between environmental-level factors and dietary intake quality in geographically isolated populations is novel and these preliminary tests of association provide an indication of the magnitude and direction of relationships, which is a necessary basis for future detailed hypothesis testing. 

## 5. Conclusions

This study is the first to empirically explore associations between modifiable community environmental-level factors and dietary intake quality for geographically isolated Australian communities. It provides important preliminary proof of concept evidence with the potential to guide future research and policies to enhance diet quality and reduce cardio-metabolic disease risk in these populations.

## Figures and Tables

**Table 1:** Associations between dietary intake features associated with cardio-metabolic disease risk and environmental-level factors.

	*n*	Discretionary Foods (% Energy)	Sugar Sweetened Beverages (% Energy)	Fruits and Vegetables (% Quantity)	Percent Estimated Average Requirement for Energy
Total (SHOP@RIC and EnRICH common communities)	13	40.6 ± 2.9	7.4 ± 1.2	9.7 ± 2.0	103.9 ± 13.1
Total (All SHOP@RIC communities)	20	41.2 ± 2.5	8.1 ± 1.7	9.3 ± 2.0	94.3 ± 18.2
Distance to neighbouring store (km)					
<82	10	42.1 ± 2.7	9.1 ± 1.6	9.0 ± 2.6	82.2 ± 15.0
≥82	10	40.3 ± 2.0	7.2 ± 1.1	9.6 ± 1.3	106.4 ± 12.1
*p*-value ^1^		0.10	<0.01	0.53	<0.001
Correlation coefficient ^2^	16	r = −0.45, *p* < 0.05	r = −0.61, *p* < 0.01	r = 0.15, *p* = 0.54	r = 0.67, *p* < 0.001
Frequency of food delivery					
Weekly	12	40.8 ± 2.6	8.3 ± 2.0	9.3 ± 2.1	93.7 ± 19.6
Fortnightly	7	42.0 ± 2.6	7.9 ± 0.9	9.2 ± 2.3	97.8 ± 16.8
*p*-value ^1^		0.32	0.69	0.92	0.61
Correlation coefficient ^2^	19	r = 0.24, *p* = 0.32	r = −0.10, *p* = 0.69	r = −0.03, *p* = 0.92	r = 0.13, *p* = 0.61
Percent Indigenous unemployment (%)					
<7.3	6	39.6 ± 2.7	7.8 ± 0.6	10.5 ± 1.2	107.1 ± 11.3
≥7.3	7	41.5 ± 2.9	7.0 ± 1.5	9.1 ± 2.5	101.2 ± 14.7
*p*-value ^1^		0.25	0.25	0.25	0.44
Correlation coefficient ^2^	13	r = 0.10, *p* = 0.75	r = −0.62, *p* = 0.02	r = −0.26, *p* = 0.38	r = −0.16, *p* = 0.61
Median Indigenous household income ($/week)					
<1187	7	40.6 ± 3.0	7.5 ± 1.0	10.8 ± 1.1	102.6 ± 9.4
≥1187	6	40.6 ± 3.0	7.1 ± 1.4	8.5 ± 2.3	105.4 ± 17.3
*p*-value ^1^		0.99	0.55	0.04	0.83
Correlation coefficient ^2^	13	r = −0.16, *p* = 0.60	r = −0.52, *p* = 0.07	r = −0.42, *p* = 0.15	r = 0.16, *p* = 0.60
Percent of dwellings needing extra bedroom/s (%)					
<60	5	39.9 ± 3.6	8.1 ± 0.7	10.8 ± 0.9	105.2 ± 11.7
≥60	8	41.0 ± 2.5	6.9 ± 1.2	9.1 ± 2.3	103.1 ± 14.6
*p*-value ^1^		0.52	0.08	0.15	0.77
Correlation coefficient ^2^	13	r = −0.36, *p* = 0.91	r = −0.55, *p* < 0.05	r = −0.39, *p* = 0.19	r = 0.07, *p* = 0.82
Percent Indigenous educated to school year 9+ (%)					
<46	6	41.4 ± 3.7	7.7 ± 1.4	10.0 ± 2.7	100.5 ± 15.5
≥46	7	39.9 ± 1.9	7.1 ± 1.0	9.5 ± 1.5	106.9 ± 11.0
*p*-value ^1^		0.35	0.37	0.65	0.39
Correlation coefficient ^2^	13	r = −0.16, *p* = 0.61	r = −0.83, *p* = 0.79	r = 0.29, *p* = 0.34	r = 0.25, *p* = 0.41

Data are means ± SD; ^1^ differences between groups (independent *t*-test); ^2^ correlation between variables (Pearson correlation). SHOP@RIC refers to the Stores Healthy Options Project in Remote Indigenous Communities study and EnRICH refers to the Environments and Remote Indigenous Cardio-Metabolic Health Project.

## Supplementary Materials

The following are available online at https://www.mdpi.com/1660-4601/16/11/1943/s1, Supplementary Materials 1: Regression model outputs for discretionary sales as a percentage of total energy, sugar-sweetened beverage sales as a percentage of total energy, fruit and vegetable sales as a percentage of total quantity and total food and beverage sales as a percentage of estimated average requirement for energy, Supplementary Materials 2: Associations between dietary intake features associated with cardio-metabolic disease risk and distance to the neighbouring store and frequency of food delivery for only the 13 common communities in both the Stores Healthy Options Project in Remote Indigenous Communities (SHOP@RIC) study and the Environments & Remote Indigenous Cardio-metabolic Health Project (EnRICH), Supplementary Materials 3: Differences between common communities in the Stores Healthy Options Project in Remote Indigenous Communities (SHOP@RIC) study and the Environments & Remote Indigenous Cardio-metabolic Health Project (EnRICH) and the non-common SHOP@RIC communities.
