# Supplementary material for: Associations between Community Environmental-Level Factors and Diet Quality in Geographically Isolated Australian Communities
Source: Int J Environ Res Public Health. 2019 May 31;16(11):1943. doi: 10.3390/ijerph16111943 (PMC6603945; doi:10.3390/ijerph16111943)
Supplement: Supplementary file 1 [file ijerph-16-01943-s001.pdf]

Supplement 1:

Regression model outputs for discretionary sales as a percentage of total energy, sugar-sweetened beverage sales as a percentage of total energy, fruit and vegetable sales as a percentage of total quantity and total food and beverage sales as a percentage of estimated average requirement for energy.

Table S1: Discretionary sales as a percentage of total energy

Source	SS	df	MS
Model	46.0097129	6	7.66828548
Residual	34.21782	2	17.10891
Total	80.2275329	8	10.0284416

Number of obs	=	9
F(6, 2)	=	0.45
Prob > F	=	0.8114
R-squared	=	0.5735
Adj R-squared	=	-0.7060
Root MSE	=	4.1363

PercentEnergyDiscretionary	Coef.	Std. Err.	t	P>|t|	Beta
Neighbouring_Store_Distance_km	-.0116275	.0403251	-0.29	0.800	-.1910715
Food_Delivery_Freq					
fortnightly	2.712725	3.112771	0.87	0.475	.4514792
Perc_Indg_UnEmp	-.187929	.4419009	-0.43	0.712	-.3971012
Med_Indig_hh_inc_wk	-.001466	.0051914	-0.28	0.804	-.1507142
Perc_dwg_need_extra_bedrm_Indig0	.186329	.2808164	0.66	0.575	.6186806
Perc_Y9_equiv_Indig	-.0640884	.1415724	-0.45	0.695	-.461741
_cons	35.82796	10.54745	3.40	0.077	.

Neighbouring_Store_Distance_km - Distance to a neighbouring store (km); Food_Delivery_Freq - Frequency of food delivery (fortnightly vs weekly); Perc_Indig_UnEmp - Percent Indigenous Australian Unemployment (%); Med_Indig_hh_inc_wk - Median Indigenous Australian household income ($/week); Perc_dwg_need_extra_bedroom_Indig0 - Percent of Indigenous Australian dwellings needing extra bedroom/s (%); Perc_Y9_equiv_Indig - Percent Indigenous Australians educated to school year 9+ (%).

Table S2: Sugar-sweetened beverage sales as a percentage of total energy

Source	SS	df	MS
Model	5.41700787	6	.902834644
Residual	2.55438337	2	1.27719168
Total	7.97139124	8	.996423904

Number of obs	=	9
F(6, 2)	=	0.71
Prob > F	=	0.6862
R-squared	=	0.6796
Adj R-squared	=	-0.2818
Root MSE	=	1.1301

PercentEnergySSB	Coef.	Std. Err.	t	P>|t|	Beta
Neighbouring_Store_Distance_km	-.0126668	.0110177	-1.15	0.369	-.6603425
Food_Delivery_Freq					
fortnightly	.7399893	.8504798	0.87	0.476	.3907078
Perc_Indg_UnEmp	.0175559	.1207374	0.15	0.898	.1176858
Med_Indig_hh_inc_wk	.0001747	.0014184	0.12	0.913	.0569831
Perc_dwg_need_extra_bedrm_Indig0	-.0967381	.0767254	-1.26	0.335	-1.01901
Perc_Y9_equiv_Indig	.0154018	.0386808	0.40	0.729	.3520347
_cons	12.80875	2.881804	4.44	0.047	.

Neighbouring_Store_Distance_km - Distance to a neighbouring store (km); Food_Delivery_Freq - Frequency of food delivery (fortnightly vs weekly); Perc_Indig_UnEmp - Percent Indigenous Australian Unemployment (%); Med_Indig_hh_inc_wk - Median Indigenous Australian household income ($/week); Perc_dwg_need_extra_bedroom_Indig0 - Percent of Indigenous Australian dwellings needing extra bedroom/s (%); Perc_Y9_equiv_Indig - Percent Indigenous Australians educated to school year 9+ (%).

Table S3: Fruit and vegetable sales as a percentage of total quantity

Source	SS	df	MS			
				Number of obs	=	9
				F(6, 2)	=	0.30
Model	18.0898849	6	3.01498081	Prob > F	=	0.8916
Residual	19.8496804	2	9.92484022	R-squared	=	0.4768
				Adj R-squared	=	-1.0928
Total	37.9395653	8	4.74244566	Root MSE	=	3.1504

PercentQuantityFandV	Coef.	Std. Err.	t	P>|t|	Beta
Neighbouring_Store_Distance_km	.0176995	.0307132	0.58	0.623	.4229463
Food_Delivery_Freq					
fortnightly	-2.551127	2.370815	-1.08	0.394	-.6174186
Perc_Indg_UnEmp	-.0485716	.3365701	-0.14	0.898	-.1492468
Med_Indig_hh_inc_wk	-.0017354	.003954	-0.44	0.704	-.2594333
Perc_dwg_need_extra_bedrm_Indig0	.0551817	.2138814	0.26	0.821	.2664385
Perc_Y9_equiv_Indig	-.0048342	.1078274	-0.04	0.968	-.050648
_cons	9.310523	8.033379	1.16	0.366	.

Neighbouring_Store_Distance_km - Distance to a neighbouring store (km); Food_Delivery_Freq - Frequency of food delivery (fortnightly vs weekly); Perc_Indig_UnEmp - Percent Indigenous Australian Unemployment (%); Med_Indig_hh_inc_wk - Median Indigenous Australian household income ($/week); Perc_dwg_need_extra_bedroom_Indig0 - Percent of Indigenous Australian dwellings needing extra bedroom/s (%); Perc_Y9_equiv_Indig - Percent Indigenous Australians educated to school year 9+ (%).

Table S4: Total food and beverage sales as a percentage of estimated average requirement for energy

Source	SS	df	MS			
Model	1498.8755	6	249.812584	Number of obs	=	9
Residual	4.92432466	2	2.46216233	F(6, 2)	=	101.46
				Prob > F	=	0.0098
Total	1503.79983	8	187.974978	R-squared	=	0.9967
				Adj R-squared	=	0.9869
				Root MSE	=	1.5691

PercentEAREnergy	Coef.	Std. Err.	t	P>|t|	Beta
Neighbouring_Store_Distance_km	.2489894	.0152976	16.28	0.004	.9450508
Food_Delivery_Freq					
fortnightly	-14.76964	1.180849	-12.51	0.006	-.5677652
Perc_Indg_UnEmp	-.781228	.1676378	-4.66	0.043	-.381287
Med_Indig_hh_inc_wk	-.0034004	.0019694	-1.73	0.226	-.0807456
Perc_dwg_need_extra_bedrm_Indig0	.1084658	.1065295	1.02	0.416	.0831851
Perc_Y9_equiv_Indig	-.2212408	.0537064	-4.12	0.054	-.3681719
_cons	100.4647	4.001242	25.11	0.002	.

Neighbouring_Store_Distance_km - Distance to a neighbouring store (km); Food_Delivery_Freq - Frequency of food delivery (fortnightly vs weekly); Perc_Indig_UnEmp - Percent Indigenous Australian Unemployment (%); Med_Indig_hh_inc_wk - Median Indigenous Australian household income ($/week); Perc_dwg_need_extra_bedroom_Indig0 - Percent of Indigenous Australian dwellings needing extra bedroom/s (%); Perc_Y9_equiv_Indig - Percent Indigenous Australians educated to school year 9+ (%).

Supplement 2:

Associations between dietary intake features associated with cardio-metabolic disease risk and distance to the neighbouring store and frequency of food delivery for only the 13 common communities in both the Stores Healthy Options Project in Remote Indigenous Communities (SHOP@RIC) study and the Environments & Remote Indigenous Cardio-metabolic Health Project (EnRICH).

	n	Discretionary Foods (% Energy)	Sugar Sweetened Beverages (% Energy)	Fruit & Vegetable (% Quantity)	Percent Estimated Average Requirement for Energy
Distance to neighbouring store (km)					
<124	6	41.0±3.6	7.9±1.0	10.4±2.7	97.5±16.0
≥124	7	40.3±2.4	6.9±1.2	9.2±1.1	109.4±7.3
P-value[1]		0.67	0.10	0.32	0.10
Correlation coefficient[2]	9	r=-0.37, p=0.33	r=-0.16, p=0.68	r=0.28, p=0.47	r=0.80, p=0.01
Frequency of food delivery					
Weekly	7	39.5±2.6	7.0±1.3	10.4±1.5	107.1±8.6
Fortnightly	6	41.9±2.8	7.8±0.9	9.0±2.4	100.1±17.1
P-value[1]		0.12	0.21	0.21	0.36
Correlation coefficient[2]	13	r=0.45, p=0.12	r=-0.37, p=0.21	r=-0.38, p=0.21	r=-0.28, p=0.36

Data are means ± SD

[1]Differences between groups (independent t-test)

[2]Correlation between variables (Pearson correlation)

Supplement 3:

Differences between common communities in the Stores Healthy Options Project in Remote Indigenous Communities (SHOP@RIC) study and the Environments & Remote Indigenous Cardio-metabolic Health Project (EnRICH) and the non-common SHOP@RIC communities.

	n	Discretionary Foods (% Energy)	Sugar Sweetened Beverages (% Energy)	Fruit & Vegetable (% Quantity)	Percent Estimated Average Requirement for Energy
Total (SHOP@RIC and EnRICH common communities)	13	40.6±2.9	7.4±1.2	9.7±2.0	103.9±13.1
Total (SHOP@RIC non-common communities)	7	42.4±1.1	9.5±1.5	8.5±1.9	76.5±11.4
Total (All SHOP@RIC communities)	20	41.2±2.5	8.1±1.7	9.3±2.0	94.3±18.2
P-value[1]		0.14	<0.01	0.19	<0.001

Data are means ± SD

[1]Differences between SHOP@RIC and EnRICH common communities and residual SHOP@RIC (non-common) communities (independent t-test).
